# Exploring interactions between women who have experienced pregnancy loss and obstetric nursing staff: a descriptive qualitative study in China

**DOI:** 10.1186/s12884-022-04787-9

**Published:** 2022-05-30

**Authors:** Jialu Qian, Weihong Wang, Shiwen Sun, Mengwei Wu, Lu Liu, Yaping Sun, Xiaoyan Yu

**Affiliations:** 1 Zhejiang University School of Medicine, NO. 268 Kaixuan Road, Hanghzhou, 310016 Zhejiang China; 2 Department of Obstetrics, Ninghai Maternal and Child Health Hospital, NO. 365 Xinghai Road, Ningbo, 315600 Zhejiang China; 3 Department of Obstetrics, Women’s Hospital School of Medicine, Zhejiang University, 1st Xueshi Road, Hangzhou, 310006 Zhejiang Province China

**Keywords:** Bereavement care, Qualitative, Pregnancy loss, Abortion, Interaction, Communication, Thematic analysis

## Abstract

**Background:**

Contradictory interactions between bereaved women who have experienced pregnancy loss and obstetric nursing staff are becoming increasingly prominent. The aim of the present study was to gain an understanding of how women who have experienced pregnancy loss and obstetric nursing staff perceive their interactions, what influencing factors impacted their experiences.

**Methods:**

A qualitative, exploratory study was conducted in a delivery room and six maternity wards of a tertiary hospital. Semi-structured interviews were performed with six nurses, 13 midwives and seven women who experienced pregnancy loss to collect rich information about how they make sense of their interactions. Thematic analysis was adopted to analyse the data.

**Results:**

Five overarching themes were identified: (1) interaction characteristics, (2) interactive contradiction, (3) influencing factors of the interaction, (4) training needs and (5) suggestions for benign interactions.

**Conclusions:**

Healthcare providers should be instructed in adopting a respectful and sympathetic attitude in communication, strengthening information support and offering patient-centred care for benign interactions. Ignoring women’s needs and using disrespectful words should be avoided. Training for preparing nurses and midwives in perinatal bereavement care and addressing heavy emotional burden is necessary. Additional efforts are needed to improve medical services and to facilitate benign interactions in induced abortion care.

**Supplementary Information:**

The online version contains supplementary material available at 10.1186/s12884-022-04787-9.

## Background

Pregnancy loss is referred to the loss of a pregnancy by miscarriage, stillbirth or termination for fetal abnormalities [1]. Miscarriage is generally defined as an unintentional pregnancy termination before foetal viability [2]. Miscarriages can be grouped into early (< 10–14 weeks) and late (> 10–14 weeks). Inevitable abortion usually develops from threatened abortion. It is vaginal bleeding with an open cervical os and viable pregnancy [3]. Globally, an estimated 23 million miscarriages occur every year [2]. The overall incidence of miscarriage is 25% [4]. Stillbirth is defined as the death of a fetus after 20 weeks gestation with a birthweight of more than 500 g. There were approximately 2.6 million stillbirths worldwide in 2015 [5]. Pregnancy terminations for foetal abnormalities including chromosomal problems, and maternal anatomic factors, immunologic factors, infection, and thrombophilia are highly stressful events [6]. In China, the reported overall incidence of foetal anomalies is 5.6%, and the termination rate of these cases is approximately 70.66% [7]. Late miscarriage, stillbirth and foetal abnormality were research emphases of this study. Because women in these cases had more interactions with nurses and midwives compared with women who experienced early pregnancy loss.

Pregnancy loss can cause destructive psychological problems [8–11], which might persist into the next pregnancy [12]. Psychological outcomes of miscarriage include increases in the risk of anxiety, depression, post-traumatic stress disorder, and suicide [2]. Women defined late miscarriage as a significant loss, a disaster, a catastrophe, or even death [13]. Stillbirth is related with a number of negative psychological symptoms including social phobia, agoraphobia, anger, a sense of failure and long-term guilt [11]. The discovery of a foetal abnormality is a shock for pregnant women and they may have psychological responses including denial of reality, reaction avoidance and self-punishing thinking [14]. In addition, the experiences of pregnancy loss may not only lead to negative psychological issues but also generate positive psychological changes. Positive psychological experiences included new life attitude, feelings of gratitude and so on [15]. Women undergoing pregnancy termination for foetal abnormality described feelings of unprecedented ease and relief of the body and mind after induced abortion [14].

It is reported that stillbirth, birth defects and mental health of pregnant women are the key issues of maternal and infant health in China [4]. In Chinese traditional culture, there is a big taboo about death. Psychological issues related to pregnancy loss are often ignored. Therefore, it has become an urgent problem to be concerned about pregnancy loss and to think about how to provide high-quality bereavement care for the women.

In China, if a definitive diagnosis of foetal death is made, abortion would be suggested to parents by doctors. For nonfatal foetal malformations, parents would sign informed consent if they decide to terminate the pregnancy. Abortion regimes are different according to women’s gestational length. Drug abortion and artificial abortion operation are applicable to women who have been pregnant within 7 weeks and for 6 ~ 14 weeks, respectively. Women are usually given an injection of Ethacridine (Rivanol) after they have been pregnant for more than14 weeks. Women have to go into labour to deliver a dead fetus in hospital [16] and their support people are not allowed to enter the labour room or delivery room. The length of their hospitalisation usually ranges from 5 to 7 days.

Studies have shown that the care parents receive around the loss has a large influence on their abilities to deal with the loss [17, 18]. However, contradictory interactions between bereaved women and obstetric nursing staff are becoming increasingly prominent [19]. In an international online survey of bereaved parents (*n* = 3769) of stillborn babies, a quarter (25.4%) of respondents reported disrespectful care and 23.5% reported disrespectful care of their baby [20]. Women having an abortion experience identified nursing care as based on physical aspects, without considering their individuality and specificities [21]. Women report that the heavy physical and mental burden of the medical staff, a lack of good communication and ineffective pain management were the main factors leading to dissatisfaction with their service [22, 23]. In contrast, nurses and midwives believe that keeping a distance from bereaved parents and focusing on nursing tasks are coping strategies they should adopt, which unfortunately adds to the distress of the parents [17, 24]. On the one hand, nurses and midwives have deficiencies in counseling and communication skills [25]. Specifically, they do not know how to use the correct expression to comfort the mother and worry that inappropriate words may upset the women further [26]. On the other hand, burnout, secondary traumatic stress and other negative emotions suggested that nurses and midwives bore a heavy emotional burden during the care process [27, 28]. The pressure to provide psychological support and the negative emotional distress of obstetric nursing staff are the main factors preventing them from providing high-quality bereavement care [29]. Therefore, they tended to adopt strategies of suppressing emotions and reduce communication with mothers [30, 31]. At present, there is no routine emotional support for the women and obstetric nursing staff in China. The majority of nurses and midwives had not received training in perinatal bereavement care (85.2%) [32].

Previous qualitative studies merely focused on women’s or healthcare providers’ broad emotional experiences [33–35]. Little attention has been given to the interaction process from both the perspectives of women and the nursing staff to comprehensively reflect the interaction issues and influencing factors in the context of pregnancy loss. Interaction refers to the process of interdependent behavior between individuals through language or other means of disseminating information. In this study, several aspects of the interaction including communication, psychological feelings, experiences of care among women who have experienced pregnancy loss and obstetrics medical staff were focused on. Appropriate communication that reflects parents’ preferences are vital for shaping women’s experience of pregnancy loss [36]. However, it is often neglected [37]. Therefore, this study aimed to explore the interactions between women who have experienced pregnancy loss and obstetric nursing staff. The findings of this study would increase our understanding of the interaction process and identify problems during the interactions, thus promoting benign interactions and improving women’s experiences in the context of perinatal bereavement care.

## Methods

### Study design

A descriptive qualitative design was used as it facilitated the interpretation of how nurses, midwives and women undergoing pregnancy termination make sense of their interactions. The consolidated criteria for reporting qualitative research (COREQ) were utilized for the study design and reporting [38]. Participants were not involved in the development of the research. Ethical approval was obtained from the Ethics Committee of the Women’s Hospital School of Medicine, Zhejiang University (IRB no. 20210091).

### Participants and recruitment

Purposive sampling was used to recruit participants at a tertiary maternity hospital in China (The normal staff-to-patient ratio is 1:10). Nurses and midwives qualified to participate if they worked in the obstetric ward or delivery room and had experiences caring for women who had experienced pregnancy loss. Women were included if they (1) were pregnant for more than 14 weeks; and (2) had already completed termination of pregnancy due to miscarriage, stillbirth or fatal foetal anomaly. Women who had a multifoetal pregnancy reduction were not included. Potential participants (nurses and midwives) were approached by a researcher (SWS), who explained the purpose of the study and asked about the participants’ willingness to participate in a confidential interview with a researcher (JLQ). Written informed consent was obtained from nurses and midwives who agreed to participate in the study. Women were telephoned after their discharge within 2 weeks. A clear explanation of the aims of the telephone interview was provided before the telephone interview and all participants were consented to the sound recording. All participants were given a pseudonym to ensure confidentiality.

### Data collection

Data collection took place between March and May 2021. Demographic and clinical data were collected prior to the interview using self-designed questionnaires. For nurses and midwives, individual interviews were conducted in a private meeting room in the hospital so that the participants would be more comfortable expressing their true feelings. For women who experienced pregnancy loss, semi-structured interviews were conducted via telephone. Interviews were conducted by JLQ, adopting a flexible topic guide (see Additional file 1). The interview script was used to generally guide the interviews. Further probing was performed according to participants’ feedback. The field notes were taken on throughout the interviews. The field notes contain information such as the interview date, non-verbal movements of the interviewees and the process of the interviews. During the interviews, researcher paid close attention to participants’ emotions. If participants had strong emotional reactions, the researcher asked them to rest a while. When serious psychological problems were noted, the researcher referred the participants to receive further psychotherapy. The interviews lasted 25–60 minutes. Interviews were digitally recorded and transcribed verbatim by JLQ. Saturation was considered to have been reached when no new themes or concepts were identified through the data collection [39].

### Data analysis

Data analysis was based on the principles of thematic analysis [40]. The analysis began as soon as the first interview was finished. The data were analysed in six stages: familiarisation with the data, generating initial codes, searching for themes, reviewing themes, defining and naming themes and producing the report. The interviews were conducted in Chinese and the transcripts were translated into English by a bilingual researcher (XYY) to maintain the linguistic accuracy. The transcripts were read multiple times to comprehend the experiences of the interviewee. Meaningful and significant basic elements were identified by highlighting every code with different colours and the initial codes were produced. Then, codes were collated into potential themes. Researchers checked if the themes work in relation to the coded extracts. After that, clear definitions and names for each theme were generated and a scholarly report of the analysis was produced. Two researchers (JLQ and WWH) independently coded the data and generated the initial subthemes and potential themes. All the authors attended the regular meetings meetings to check the uncertain themes and ensure the confirmability of the interpretations, and any disagreement was discussed until a consensus was reached [41].

To improve the rigour, the field notes were examined during the data analysis to better understand the data. The researchers remained reflexivity to recognise their potential impact on the study findings and maintained faithful to the perspectives of the interviewees. After data analysis, the researchers telephoned six interviewees (2 midwives, 2 nurses and 2 women) and openly shared the research results with them. If the interviewees disagree with the findings, the researchers then rechecked the relevant codes to work on the final analysis. In this way, the research team got feedback from the interviewees to ensure the accuracy and credibility of the results. Six interviewees considered that the research results represented their perceptions and no important themes were missed. Two authors (MWW and LL) monitored the entire process of data analysis.

## Results

### Characteristics of the participants

Interviews were conducted with a total of 26 participants (6 nurses, 13 midwives, 7 women who had experienced pregnancy loss). The mean age of the nurses and midwives was 31 years, with a range from 24 to 51 years. The nurses and midwives reported a range of 1–33 years of clinical experience in nursing. Most of the nurses and midwives had a baccalaureate degree, and only 1 midwife had a master’s degree. Ten nurses and midwives were married, and 9 were unmarried. Seven of the ten married nurses and midwives had children. Only one nurse had received perinatal bereavement care education. The mean age of the women who experienced a pregnancy loss was 31 years, range 24–37 years. All of the women had a baccalaureate degree and above. Two women had previously given birth and five were primiparas. The gestational age of the women ranged from 15 to 28 weeks. The characteristics of the interviewees are outlined in Tables 1 and 2.

**Table 1:** Characteristics of midwives and nurses

Variable	Category of variable	Number
Occupation	Midwife	13
	Nurse	6
Age	< 35 years	14
	≥ 35 years	5
Years of clinical experience	≤ 5 years	10
	6–10 years	4
	11–20 years	3
	> 20 years	2
Education level	Undergraduate	18
	Postgraduate	1
Marital status	Married	10
	Unmarried	9
Do you have a child?	Yes	7
	No	12
Have you received any perinatal bereavement care education?	Yes	1
	No	18

**Table 2:** Characteristics of women who have experienced pregnancy loss

Variable	Category of variable	Number
Age	< 35 years	5
	≥ 35 years	2
Education level	Junior college	3
	Undergraduate	3
	Postgraduate	1
Gravidity	1	2
	2	5
Parity	0	5
	1	2
Gestational age	15–20 weeks	2
	20–28 weeks	5
Diagnosis	Fatal foetal anomaly	4
	Inevitable abortion	2
	Stillbirth	1

Five overarching themes emerged from the analysis and were supported by fifteen subthemes (Table 3), which are discussed below with illustrative extracts from the interviews. A detailed description of the data coding is available in Additional file 2.

**Table 3:** Themes and subthemes

Themes	Definition of each theme	Subthemes
Interaction characteristics	This theme described approaches nursing staff adopted and womem’s features during the interactions. Their interaction process could have psychological effects on both nursing staff and the women.	Approaches to interaction
		The sensitive and grieving women
		Mutual influence
Interactive contradiction	This theme described situations that may lead to unpleasant interaction experiences. Ignoring women’s needs and using disrespectful words are common causes.	Being ignored
		Disrespectful words
Influencing factors of interactions	This theme pointed out that external and internal factors could have impact on the interactions. Heavy clinical workload was viewed as an external factor. Ability and awareness level, emotional experiences of nurses and midwives were considered as internal factors.	Heavy clinical workload
		Lack of ability and awareness
		Emotional experiences of nurses and midwives
Training needs	This theme referred to a situation where the participants recognised inadequacies with regards to clinical professional knowledge and humanistic care skills among nursing staff. The necessity of organising professional training was proposed.	Clinical professional knowledge and skills
		Humanistic care skills
Suggestions for benign interactions	This theme suggested that efforts should be made on better medical environment for the women, more effective personnel management, better bereavement services and preparing competent nursing professionals for achieving benign interactions in the context of pregnancy loss.	Improvement of the medical environment and management
		Optimization of bereavement care
		Preparing competent nursing professionals

### Theme 1: interaction characteristics

#### Approaches to interaction

Some nurses and midwives applied a respectful communication approach to their interaction with the women. Sensitive words were avoided, and euphemisms were chosen to protect the women. Nurses and midwives put themselves in the women’s position to help them understand what they were going through. Positive words were delivered to the women via downward comparisons and rebuilding the women’s hope.

*“I always tell women that although this is a big deal in your life, you still have to look forward. I would take some examples of others to make them feel that they were not the most special. When a woman feels that someone is worse than her, she will feel slightly relieved.” [Vivian; Midwife]*

Nurses and midwives provided sufficient information support through adequate health education and targeted instructions (Additional file 3, Quote 1). Nurses and midwives claimed that providing “woman-centred care” was important and they had to be very familiar with the conditions of the women who experienced pregnancy loss. They tried to satisfy the women’s needs and give timely nursing interventions during the pregnancy termination (Quote 2).

A task-centred approach was adopted by some nurses and midwives. The interaction was established only to convey short messages to prepare the women for the abortion. The women agreed that the interaction was limited and did not cover any aspects of psychological care (Quote 3). Another situation was that nurses and midwives considered their comfort to women useless and that it might even induce negative emotions in the women. Additionally, the nurses and midwives reported that they provided care based on their personal experience without any theoretical guidance or taking the women’s needs into consideration. The above circumstances make health care providers passive during interactions.

*“I don’t think there is any theoretical guidance. It’s empiricism. I make subjective judgement according to my personal experience instead of truly understanding women’s needs.” [Sarah; Midwife]*

#### The sensitive and grieving women

The women were mostly described as sensitive during the interaction. They were easily angered by care providers’ casual words.

*“A midwife had a bad attitude when I asked for help with contractions; she gave me the feeling that you're here to terminate pregnancy. Do not disturb me taking care of other pregnant women.” [Doris; Women]*

Due to their negative emotions, different women behaved differently. Some women had wild mood swings. Emotional flooding, anxious emotions, and self-accusation were commonly seen. Some women remained silent and were not willing to express their needs. They responded to medical staff coldly (Quote 4).

#### Mutual influence

The care provided by nurses and midwives might influence the women’s psychological recovery since the women were quite sensitive to the healthcare providers’ words and behaviour during the abortion (Quote 5). On the other hand, the healthcare providers’ moods were easily affected by the women’s traumatic experiences. Nurses and midwives felt scared and guilty. Sometimes they may have had nightmares and were reluctant to deliver the foetuses.

*“One night, i approached 5 women who had induced abortion. My mood was very depressed. When I closed my eyes, there were several infant cadavers in my mind. It is easy to have nightmares.” [Sarah; Midwife]*

### Theme 2: interactive contradiction

#### Being ignored

Being ignored was the most frequently mentioned complaint. Women who experienced pregnancy loss desired more attention from nurses and midwives. Instead, compared with normal pregnant women, they were neglected in clinical practice because the medical staff do not have to monitor the foetal heartbeat regularly. These circumstances are likely to trigger interactive contradictions.

*“Few medical staff will come to the women to touch their contractions and comfort them. When women suffer from contractions, they feel that no one care about them.” [Vivian; Midwife]*

#### Disrespectful words and attitude

Although the nurses and midwives did not mean to cause offence, sometimes they communicated with the women without adopting a sensitive attitude. Some words were misunderstood by the women, and they might conclude that the medical staff did not respect them (Quote 7). Some of nurses and midwives were abrupt and aloof. They were impatient with satisfying the women’s needs. Women reported that they were afraid of expressing their needs due to impatient attitude of nursing staff.

*“The midwife’s attitude was blunt and impatient. The midwife asked me to tolerate the pain. Even though my pain was severe, I didn't dare to call a nurse” [Jane; Woman]*

### Theme 3: influencing factors of interactions

#### Heavy clinical workload

Clinical work is quite busy, and human resources are deficient. Nurses and midwives reported that they did not have enough time to provide psychological care for these women, which significantly affected the quality of the communication and time spent with the women (Quote 8).

In addition, the emphasis of obstetric work is to ensure the safety of mothers and their babies. For women who experience a pregnancy loss, nurses and midwives do not need to ensure the safety of the foetus; therefore, the attention given to these women is reduced to some extent.

*“If many pregnant women are going to give birth, their babies must be taken care of immediately because their babies are alive. In your limited time, you definitely value lived babies. Therefore, when we are very busy, we will take care of the normal mothers first. The women who terminated their pregnancy will be ranked behind. It is a reality.” [Sarah; Midwife]*

#### Lack of ability and awareness

Nurses and midwives described that they lacked sufficient communication skills, psychological skills and professional medical knowledge related to pregnancy loss, which resulted in less communication with these women. Young nurses and midwives had difficulty understanding those women’s emotions because they had not experienced pregnancy and the feeling of becoming a mother (Quote 9). Due to insufficient clinical work experience, they may consider these women to be relatively safe and spend most of their time with healthy pregnant women.

*“In fact, those women truly require attention. Many of them often suffer from high fever and septic shock. Young nurses and midwives have never encountered these cases, so they do not have such awareness.” [Vivian; Midwife]*

#### Emotional experiences of nurses and midwives

Emotional exhaustion and burnout were considered resistance factors of benign interactions. Nurses and midwives reported that when they first started working, they were sympathetic to the women. However, as time went by, they gradually became numb when faced with this patient population due to heavy and continuous emotional burden (Quote 10). In contrast, some nurses and midwives were sympathetic to the women, which facilitated the provision of better care.

*“I sympathize with this unborn child and the mother, and I will do better during the process.” [Vivian; Midwife]*

### Theme 4: training needs

#### Clinical professional knowledge and skills

Nurses and midwives claimed that they wanted to learn more about pain management skills. Women who terminated a pregnancy had to endure a more severe degree of pain than women undergoing normal childbirth. Nurses and midwives felt powerless when it came to relieving the women’s pain.

*“I will guide women to breathe when they feel painful, but it seems no analgesic effect. Sometimes I feel quite powerless.” [Anna; Nurse]*

Some experienced nurses and midwives proposed that nursing staff should study relevant medical knowledge instead of being confined to nursing knowledge. They also need to possess the capacity to answer relevant questions raised by the women to facilitate their trust (Quote 12).

#### Humanistic care skills

The nurses and midwives mentioned that they were willing to learn communication and psychological support skills for women undergoing pregnancy termination. They would like to learn specific content and key points to use during communication based on the women’s psychological characteristics (Quote 13).

### Theme 5: suggestions for benign interactions

#### Improvement of the medical environment and management

The nurses, midwives and women all suggested that a private room should be provided for this population to reduce environmental impacts on their moods (Quote 14). Additionally, the midwives hoped they could obtain emotional support from their colleagues and managers. During the delivery process, they wanted a colleague to accompany them. Additional personnel is also an important aspect, especially in the delivery room.

*“There are still too few staff in the delivery room, so I hope the hospital can arrange more staff here. We were often alone in the predelivery room. In my opinion, there should be at least two staff in the predelivery room.” [Julia; women]*

#### Optimization of bereavement care

The midwives thought that the current practices could not truly satisfy women, and they did not know how to deal with the baby to show their respect and just adopted routine procedures, which might hurt women.

*“When showing the stillborn baby to the mother, we put the baby in a basin. This indicates that we don’t fully respect this life. The woman would feel crueler as a mother. I think we should dress the baby or cover it a little.” [Sarah; midwife]*

Family support is proposed to be extremely important to these women. The midwives suggested that it would be better if family members could accompany the women before or after delivery. Due to the busy clinical work and insufficient personnel, the women were usually completely alone after delivery, which might make them feel helpless and abandoned. Timely responses to the women’s needs and providing sympathetic company to these women need to implemented (Quote 15).

#### Preparing competent nursing professionals

Nursing professionals should be prepared for possessing the core competence of facilitating benign interactions. Reflection and summarizing their clinical experiences have been proposed to be important processes of accumulating practical benign interaction experience (Quote 16). Transpositional consideration is helpful for medical staff to understand and care about these women in a sympathetic and respectful way (Quote 17).

## Discussion

### Main findings

Our study showed that different approaches used by nursing professionals in interactions might lead to different outcomes. Generally, women were sensitive and distressing during the interactions. The interaction process could have both impact on psychological well-being of providers and the women. Ignoring women’s needs and using disrespectful words were main reasons for interaction contradiction. Heavy clinical workload, lack of ability and awareness and the heavy emotional burden on the nursing staff were the main influencing factors of interactions. Training needs for clinical professional skills and humanistic care skills were highlighted. Some helpful suggestions were proposed to facilitate benign interactions including improvement of the medical environment and management, optimization of bereavement care and competent nursing professionals.

### Comparison with literature

Respectful communication is crucial for this sensitive population, which is consistent with the framework and principles of the practice of respectful and supportive perinatal bereavement care [42, 43]. Good communication is the issue most often mentioned in studies of parents’ experiences of bereavement care [44, 45]. In our study, it was suggested that healthcare staff should show their sympathy and patience with a sensitive attitude. It could provide a feeling of support so that women are more able to adapt to the crisis. Moreover, women-centred care and providing adequate information are also important aspects of benign interaction. These results are similar to previous findings [37, 46, 47]. In contrast, task-based communication, disrespectful words, neglecting women’s needs and delayed responses may lead to undesirable interactions and even conflicts, which should be avoided. The emphasis on women’s needs varied in different stages [14, 48]. Therefore, prompt feedback and targeted care should be given to women based on their needs and psychological responses.

In regard to influencing factors of interactions, a heavy workload and insufficient human resources could significantly affect the quality of bereavement care, which is similar to previous findings [25, 49]. It is necessary to add personnel and provide equal care to all women. Burnout, emotional exhaustion and other negative emotions among healthcare staff may lead to reduced quality of care [17], and sufficient emotional support should be offered to relieve their emotional labour [50]. There is a need for employee assistance programmes, including group psychological interventions and meditation, to relieve negative moods [27, 51, 52].

Previous studies showed that the highest quality of bereavement care could be guaranteed by providing comprehensive and ongoing training for healthcare staff [53–55]. Hence, training nurses and midwives in perinatal bereavement care is necessary [56]. In this study, lack of ability and awareness were influencing factors of interactions. Cultivating healthcare staff’s awareness of caring for women with sympathy and respect is important and should be included in the training. Insufficient treatment of pain and dissatisfaction with pain management were commonly seen during abortions [23]. Effective pain management strategies [57–60] and continuous professional attendance [61] should be provided to women to cope with the pain and improve their satisfaction with the overall experience. Requirements of receiving training about clinical professional knowledge and humanistic care skills were put forward. More dialogue with psychologists and education on the causes of pregnancy loss could improve nursing staff’s professional knowledge and enhance the women’s trust in them [50]. In terms of humanistic care skills, active listening and responding empathically to women are useful skills that could be adopted by nursing staff [47, 62, 63].

For the medical environment, providing a private space to accelerate the women’s feelings of comfort and to enable providers to satisfy women’s needs is necessary [62]. In regard to abortion services, uniform training related to bereavement care strategies such as seeing the baby and making memories should be offered to providers [47]. Instructions should be given to families to enhance family support for these vulnerable women [64, 65] because family support is the most significant support required by women who have lost babies [66]. Integrating partners into abortion care to enhance the quality of companions during the entire process is worth consideration [67]. During the interview, we found that nurses and midwives who had children were more likely understand women’s experiences and emotional pain. Nursing staff without childbirth experience felt a little difficult to express their sympathy. Therefore, training for healthcare providers with little clinical experience of pregnancy loss, especially those with no kids, is a priority. They are in greater needs of receiving training to guide their practice. Our findings showed that nurses and midwives should learn to provide respectful care via introspecting their practice. Because reflection (e.g. writing or talking with colleagues) can provide us with insights that bring clarity and wisdom [68].

### Implications for hospital policies

First, provider training should be regularly organised to guide obstetric nursing staff how to provide sensitive and respectful care for women who have experienced pregnancy loss. Our findings including approaches to interactions, characteristics of the grieving women, approaches of avoiding interaction contradiction should be integrated into the training. Nurses’ and midwives’ learning needs including clinical professional knowledge and humanistic care skills should be satisfied. Second, hospitals also need to pay close attention to the mental health of providers. Providing sufficient support for obstetrics medical staff via providing establishing professional psychological support team in hospitals could be considered. More effective human resources management can be useful to ease the heavy workload. Thereby, nurses and midwives could provide high-quality bereavement care for the women in good mental state. Last but not least, it is important to forming systematic and standardized bereavement care policies covering information support, emotional support, communication and so on. Intimate service such as providing private space for the bereaved women could be offered in bereavement care.

### Strengths and limitations

The COREQ checklist was used for the study design and reporting, ensuring the quality and rigour of this qualitative study. A representative sample of stakeholders guaranteed a wide range of data sources from which to draw conclusions. Our study not only focused on interaction conflicts and clinical deficiencies but also paid attention to benign interaction experiences. This was helpful to provide enlightenment about improving clinical services and future training for medical staff during pregnancy termination.

A limitation of this study is that nurses and midwives might be cautious when they describe conflicts during an interaction, although we explained that the interview was anonymous. This may have an impact on thoroughly exploring conditions of interactive contradictions. Although researchers interviewed 13 midwives and 7 nurses until saturation was reached, the perceptions expressed may not be representative of these communities as a whole. Besides, we only interviewed women who were pregnant for more than 14 weeks for gaining more information of their interaction experiences. Women experiencing early pregnancy loss (< 14 weeks) have been excluded, which may influence the representativeness of our findings to some extent.

## Conclusions

In conclusion, healthcare providers should be instructed in adopting a respectful and sympathetic attitude, strengthening information support and offering patient-centred care for benign interactions. Ignoring women’s needs and using disrespectful words are taboos among interactions with women who have experienced pregnancy loss. Perinatal bereavement care training with the aim of increasing healthcare providers’ awareness of humanistic care provision, clinical competency enhancement and heavy emotional burden relief are necessary. Constant effort is still needed to improve medical services and facilitate benign interactions in induced abortion care.

## Supplementary Information

**Additional file 1.** Interview guide

**Additional file 2.** Data coding: theme, subtheme and substantive codes

**Additional file 3.** Themes and results

## Data Availability

The datasets analysed during the current study are available from the corresponding author on reasonable request.

## Abbreviation

COREQ: Consolidated criteria for reporting qualitative research

## References

[1] Robinson GE (2014). Pregnancy loss. Best Pract Res Clin Obstet Gynaecol.

[2] Quenby S, Gallos ID, Dhillon-Smith RK, Podesek M, Stephenson MD, Fisher J, Brosens JJ, Brewin J, Ramhorst R, Lucas ES, McCoy RC, Anderson R, Daher S (2021). Miscarriage matters: the epidemiological, physical, psychological, and economic costs of early pregnancy loss. Lancet..

[3] Redinger A, Nguyen H (2022). Incomplete abortions.

[4] Qiao J, Wang Y, Li X, Jiang F, Zhang Y, Ma J, et al. A lancet commission on 70 years of women's reproductive, maternal, newborn, child, and adolescent health in China. Lancet. 2021. 10.1016/S0140-6736(20)32708-2.

[5] Lawn JE, Blencowe H, Waiswa P, Amouzou A, Mathers C, Hogan D, Flenady V, Froen JF, Qureshi ZU, Calderwood C, Shiekh S, Jassir FB, You D (2016). Stillbirths: rates, risk factors, and acceleration towards 2030. Lancet..

[6] Michels TC, Tiu AY (2007). Second trimester pregnancy loss. Am Fam Physician.

[7] Wu Y, Cheng W (2016). Birth defect profile and prenatal screening. Chinese J Fam Plan Gynecotokol.

[8] Haagen JFG, Moerbeek M, Olde E, Hart OVD, Kleber RJ (2015). PTSD after childbirth: a predictive ethological model for symptom development. J Affect Disord.

[9] Sun S, Yang M, Zhang J, Zhou X, Jia G, Yu X (2020). Family support for pregnant women with foetal abnormality requiring pregnancy termination in China. Health Soc Care Community.

[10] Krosch DJ, Shakespeare-Finch J (2017). Grief, traumatic stress, and posttraumatic growth in women who have experienced pregnancy loss. Psychol Trauma Theory Res Pract Policy.

[11] Burden C, Bradley S, Storey C, Ellis A, Heazell AE, Downe S, Cacciatore J, Siassakos D (2016). From grief, guilt pain and stigma to hope and pride - a systematic review and meta-analysis of mixed-method research of the psychosocial impact of stillbirth. BMC Pregnancy Childbirth.

[12] Fernández Ordóñez E, Rengel Díaz C, Morales Gil IM, Labajos Manzanares MT (2018). Post-traumatic stress and related symptoms in a gestation after a gestational loss: narrative review. Salud Ment.

[13] Kukulskienė M, Žemaitienė N. Experience of late miscarriage and practical implications for post-Natal health care: qualitative study. Healthcare (Basel). 2022;10(1). 10.3390/healthcare10010079 PMID:35052243. 10.3390/healthcare10010079 PMC8775379 35052243

[14] Qian J, Sun S, Yang M, Zhou X, Wu M, Yu X (2020). Psychological trajectories of Chinese women undergoing pregnancy termination for fetal abnormality: a descriptive qualitative study using expressive writing. J Clin Nurs.

[15] Zhou X, Yang M, Qian J, Sun S, Wu M, Xiao Y, Yu X (2019). Women's experiences of termination of pregnancy for a fetal anomaly during the postpartum period:a qualitative study. Chin J Nurs.

[16] Jinhong X (2017). Effect of mifepristone combined with rivanol in mid-term labor induction. J Clin Med Liter.

[17] Ellis A, Chebsey C, Storey C, Bradley S, Jackson S, Flenady V, Heazell A, Siassakos D (2016). Systematic review to understand and improve care after stillbirth: a review of parents’ and healthcare professionals’ experiences. BMC Pregnancy Childbirth.

[18] Claudia R, Miriam L, Elena A, Gianpaolo R, Marco B, Roberto B, Alfredo V (2018). Stillbirth and perinatal care: are professionals trained to address parents' needs?. Midwifery..

[19] Mills TA, Ricklesford C, Cooke A, Heazell AE, Whitworth M, Lavender T (2014). Parents' experiences and expectations of care in pregnancy after stillbirth or neonatal death: a metasynthesis. BJOG..

[20] Atkins B, Blencowe H, Boyle FM, Sacks E, Horey D, Flenady V. Is care of stillborn babies and their parents respectful? Results from an international online survey. BJOG. 2022. 10.1111/1471-0528.17138 PMID:35289061. 10.1111/1471-0528.17138 35289061

[21] Mariutti MG, Almeida AMD, Panobianco MS (2007). Nursing care according to women in abortion situations. Rev Lat Am Enferm.

[22] Hu H, Qin C, Tang S, Deng Y, Li Y, Gong N (2020). Analysis of potential factors of healthcare violence from the perspective of patients with fetal malformations inducing labor based on qualitative research. J Shaoyang Univ.

[23] Georgsson S, Carlsson T (2019). Pain and pain management during induced abortions: a web-based exploratory study of recollections from previous patients. J Adv Nurs.

[24] Lee C (2012). 'She was a person, she was here': the experience of late pregnancy loss in Australia. J Reprod Infant Psychol.

[25] Mauri PA, Squillace F (2017). The experience of Italian nurses and midwives in the termination of pregnancy: a qualitative study. Eur J Contracep Repr.

[26] Doherty J, Cullen S, Casey B, McMahon A, Brosnan M, Sheehy L, Lloyd B, Barry T, Coughlan B (2017). “I'm afraid of upsetting them further”: student midwives educational needs in relation to bereavement in the maternity setting. BMC Pregnancy Childb.

[27] Teffo ME, Levin J, Rispel LC (2018). Compassion satisfaction, burnout and secondary traumatic stress among termination of pregnancy providers in two south African provinces. J Obstet Gynaecol Re.

[28] Zaręba K, Banasiewicz J, Rozenek H, Ciebiera M, Jakiel G (2020). Emotional complications in midwives participating in pregnancy termination procedures—polish experience. Int J Env Res Pub He.

[29] Garel M, Etienne E, Blondel B, Dommergues M (2010). French midwives' practice of termination of pregnancy for fetal abnormality. At what psychological and ethical cost?. Prenatal Diag.

[30] Chen FH, Hu WY (2013). The impact of perinatal death on nurses and their coping strategies. J Nurs.

[31] Mizuno M (2011). Confusion and ethical issues surrounding the role of Japanese midwives in childbirth and abortion: a qualitative study. Nurs Health Sci.

[32] Qian J, Wu H, Sun S, Wang M, Yu X (2022). Psychometric properties of the Chinese version of the perinatal bereavement care confidence scale (C-PBCCS) in nursing practice. PLoS One.

[33] Qian J, Sun S, Yang M, Zhou X, Wu M, Yu X (2020). Psychological trajectories of Chinese women undergoing pregnancy termination for foetal abnormality: a descriptive qualitative study using expressive writing. J Clin Nurs.

[34] Armour S, Gilkison A, Hunter M (2018). The lived experience of midwives caring for women facing termination of pregnancy in the late second and third trimester. Women Birth.

[35] Yang CF, Che HL, Hsieh HW, Wu SM (2016). Concealing emotions: nurses' experiences with induced abortion care. J Clin Nurs.

[36] Smith LK, Dickens J, Bender AR, Bevan C, Fisher J, Hinton L (2020). Parents' experiences of care following the loss of a baby at the margins between miscarriage, stillbirth and neonatal death: a UK qualitative study. BJOG..

[37] Austin L, Littlemore J, McGuinness S, Turner S, Fuller D, Kuberska K (2021). Effective communication following pregnancy loss: a study in England. Camb Q Healthc Ethics.

[38] Tong A, Sainsbury P, Craig J (2007). Consolidated criteria for reporting qualitative research (COREQ): a 32-item checklist for interviews and focus groups. Int J Qual Health Care.

[39] Trotter RN (2012). Qualitative research sample design and sample size: resolving and unresolved issues and inferential imperatives. Prev Med.

[40] Braun V, Clarke V (2006). Using thematic analysis in psychology.

[41] Barbour RS (2001). Checklists for improving rigour in qualitative research: a case of the tail wagging the dog?. BMJ..

[42] Boyle FM, Horey D, Middleton PF, Flenady V (2020). Clinical practice guidelines for perinatal bereavement care — an overview. Women. Birth..

[43] Sands Australia. Sands Australian principles of bereavement Careed^e. Melbourne; 2018.

[44] Hodgson J, Pitt P, Metcalfe S, Halliday J, Menezes M, Fisher J, Hickerton C, Petersen K, McClaren B (2016). Experiences of prenatal diagnosis and decision-making about termination of pregnancy: a qualitative study. Aust N Z J Obstet Gynaecol.

[45] Asplin N, Wessel H, Marions L, Georgsson ÖS (2014). Pregnancy termination due to fetal anomaly: women's reactions, satisfaction and experiences of care. Midwifery..

[46] Xiaoli Z, Xiaoyan Y (2020). Advances progress on supportive care needs of women with fetal abnormalities. Chin J Nurs.

[47] Smith LK, Dickens J, Bender Atik R, Bevan C, Fisher J, Hinton L (2020). Parents’ experiences of care following the loss of a baby at the margins between miscarriage, stillbirth and neonatal death: a UK qualitative study. BJOG Int J Obstet Gynaecol.

[48] Furtado-Eraso S, Escalada-Hernández P, Marín-Fernández B (2020). Integrative review of emotional care following perinatal loss. West J Nurs Res.

[49] Parker A, Swanson H, Frunchak V (2014). Needs of labor and delivery nurses caring for women undergoing pregnancy termination. J Obstet Gynecol Neonatal Nurs Jognn.

[50] Qian JL, Pan PE, Wu MW, Zheng Q, Sun SW, Liu L, Sun YP, Yu XY (2021). The experiences of nurses and midwives who provide surgical abortion care: a qualitative systematic review. J Adv Nurs.

[51] Kim JI, Yun JY, Park H, Park SY, Ahn Y, Lee H, Kim TK, Yoon S, Lee YJ, Oh S, Denninger JW, Kim BN, Kim JH (2018). A Mobile videoconference-based intervention on stress reduction and resilience enhancement in employees: randomized controlled trial. J Med Internet Res.

[52] Qinyu W, Liping S, Jingxin W (2019). The study of the function of group psychological intervention of Balint group on nurses of self-efficacy and emotional labor. J Xinjiang Med Univ.

[53] Bereavement Care Standards Group. National Standards for bereavement care following pregnancy loss and perinatal Deathed^e. Dublin: Health Service Executive; 2016.

[54] Peters MDJ, Lisy K, Riitano D, Jordan Z, Aromataris E (2015). Caring for families experiencing stillbirth: evidence-based guidance for maternity care providers. Women Birth.

[55] Shakespeare C, Merriel A, Bakhbakhi D, Blencowe H, Boyle FM, Flenady V, Gold K, Horey D, Lynch M, Mills TA, Murphy MM, Storey C, Toolan M (2020). The RESPECT study for consensus on global bereavement care after stillbirth. Int J Gynecol Obstet.

[56] Qian J, Sun S, Wu M, Liu L, Yaping S, Yu X (2021). Preparing nurses and midwives to provide perinatal bereavement care: a systematic scoping review. Nurs Educ Today.

[57] Constant D, Tolly KD, Harries J, Myer L (2014). Mobile phone messages to provide support to women during the home phase of medical abortion in South Africa: a randomised controlled trial. Contraception..

[58] Mecdi KM, Aslan E (2021). Efficacy of nursing support in the pre- and postmedical termination of pregnancy phases: a randomized study. Omega (Westport)..

[59] Zhang YN, Jin-Jin YU, Jin-Ping LI (2007). Application of psychological intervention evaluated by symptom checklist 90 on women accepting artificial abortion. Chin J Clin Psychol.

[60] Jackson E, Kapp N (2020). Pain Management for Medical and Surgical Abortion between 13 and 24 weeks gestation: a systematic review. BJOG Int J Obstet Gynaecol.

[61] Boryri T, Noori N, Mohammad T, Alireza Y, Fariba. (2016). The perception of primiparous mothers of comfortable resources in labor pain (a qualitative study). Iran J Nurs Midwifery Res.

[62] Strefling IDSS, Lunardi Filho WD, Kerber NPDC, Soares MC, Ribeiro JP (2015). Nursing perceptions about abortion management and care: a qualitative study. Texto Contexto Enfermagem.

[63] Banerjee SC, Manna R, Coyle N, Penn S, Gallegos TE, Zaider T, Krueger CA, Bialer PA, Bylund CL, Parker PA (2017). The implementation and evaluation of a communication skills training program for oncology nurses. Transl Behav Med.

[64] Sun S, Mengye Y, Zhang J, Zhou X, Jia G, Yu X (2020). Family support for pregnant women with foetal abnormality requiring pregnancy termination in China. Health Soc Care Comm.

[65] Sun S, Li J, Ma Y, Bu H, Luo Q, Yu X (2017). Effects of a family-support programme for pregnant women with foetal abnormalities requiring pregnancy termination: a randomized controlled trial in China. Int J Nurs Pract.

[66] Allahdadian M, Irajpour A, Kazemi A, Kheirabadi G (2015). Social support: an approach to maintaining the health of women who have experienced stillbirth. Iran J Nurs Midwifery Res.

[67] Shiwen S (2018). Family support status and intervention studyof pregnant women with fetal abnormality requiring pregnancy termination ed^e.

[68] White KR (2022). To learn: the power of reflection. Nurs Outlook.

