# Supplementary material for: Exploring interactions between women who have experienced pregnancy loss and obstetric nursing staff: a descriptive qualitative study in China
Source: BMC Pregnancy Childbirth. 2022 May 30;22:450. doi: 10.1186/s12884-022-04787-9 (PMC9153172; doi:10.1186/s12884-022-04787-9)
Supplement: Supplementary file 1 — Additional file 1. Interview guide [file 12884_2022_4787_MOESM1_ESM.doc]

**Additional file 1 Interview guide**

**Interview guide for nurses and midwives**

- What do you pay special attention to in the process of communicating with women who have experienced pregnancy loss?
- Have you ever had any conflict with these women?
- Do you have any experience to share on how to avoid conflict during interaction?
- Are you comfortable and satisfied with current interaction with these women?
- What factors would affect the quality of your interaction?
- How do you rate your ability to interact with the bereaved women? What skills need to be improved?
- Are there any deficiencies in the clinical care for this population?
- Do any suggestions for improving interaction quality with these women?

**Interview guide for women who have experienced pregnancy loss**

- Are you satisfied with the quality of interaction you experienced with nurses and midwives and why?
- Are nurses and midwives involved in your psychological care?
- Is there anything that makes you particularly unpleasant in the interaction process?
- What factors do you think affect the quality of your interaction?
- Are there any nurses or midwives that impress you and why?
- In what way would you like nurses and midwives to interact with you?
- What ability do you want nurses and midwives to improve during the interaction?
