# Supplementary figures and images for: Exploring interactions between women who have experienced pregnancy loss and obstetric nursing staff: a descriptive qualitative study in China
Source: BMC Pregnancy Childbirth. 2022 May 30;22:450. doi: 10.1186/s12884-022-04787-9 (PMC9153172; doi:10.1186/s12884-022-04787-9)

**Additional file 2 Data coding: theme, subtheme and substantive codes**


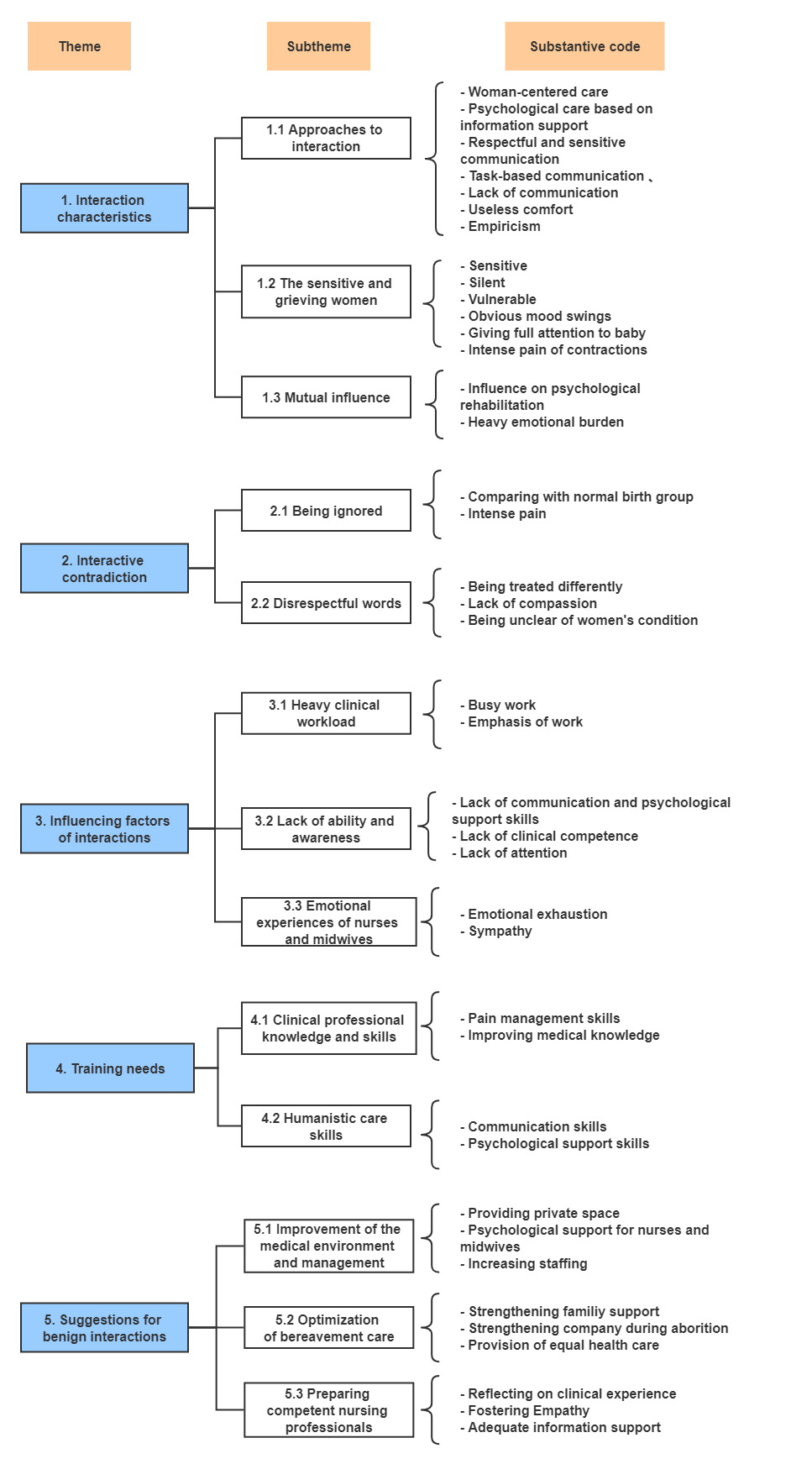

Supplement: Supplementary file 2 — Additional file 2. Data coding: theme, subtheme and substantive codes [file 12884_2022_4787_MOESM2_ESM.doc]
