# Supplementary material for: Exploring interactions between women who have experienced pregnancy loss and obstetric nursing staff: a descriptive qualitative study in China
Source: BMC Pregnancy Childbirth. 2022 May 30;22:450. doi: 10.1186/s12884-022-04787-9 (PMC9153172; doi:10.1186/s12884-022-04787-9)
Supplement: Supplementary file 3 — Additional file 3. Themes and results [file 12884_2022_4787_MOESM3_ESM.doc]

**Additional file 3 Themes and results**

| **Themes** | **Subthemes** | **Example quotes** |
| --- | --- | --- |
| Interaction characteristics | Approaches to interaction | 1. *“ You can tell her some help she can get in the follow-up, how long will it take to recover in the later period, and what needs to be paid attention to in postpartum period, so that she can be more at ease.” [Nina; Midwife]* 2. *“If a woman feels painful contractions, we should give her some measures to let her know that you care about her. If your expression or language is cold, it will aggravate her emotional collapse. If she has a request to dress and take pictures for the baby, I will try to satisfy her needs.” [Claire; Midwife]* 3. *“Most of us mainly ensure women’s safety. This population is special, so most of the medical staff will be more cautious. We only talk about treatment and do not mention other things.” [Ashley; Nurse]* |
|  | Interaction characteristics of the bereaved women | 1. *“For those women, you ask her one sentence, and then she answers one sentence. They don't ask many questions. Maybe they endured the pain silently in the early stage, and was silent again after delivery.” [Jody; Midwife]* |
|  | Mutual influence | 1. *“Childbirth is one of the deepest memories in a woman's life. Even though my daughter is nine years old, I still remember my delivery clearly. Induced abortion is a bad experience. If we are not very concerned about those women, they may remember our bad behavior for a lifetime, which will also affect their psychological recovery.” [Rose; Midwife]* |
| Interactive contradiction | Being ignored | 1. *“The main complaint we may encounter most is that women think no medical staff take care of them, especially when we are busy in the delivery room. I may take charge of 2 rooms. If the conditions of those women are fine, I may go to the other room. And there may be nobody in the room where she is. This will cause the mother's psychological gap.This is a potential contradiction during the interaction.” [Claire; Midwife]* |
|  | Disrespectful words | 1. *“I asked a midwife if I could go to the toilet? She said yes. But another midwife told me not to get out of bed and go to the toilet before. She replied: ‘Your baby is different from others.’ I was really angry at that time.” [Daisy; Woman]* |
| Influencing factors of interactions | Heavy clinical workload | 1. *“ Clinical work is really too busy. It is difficult for me to take care of those women’s mental health. When a woman in the next room is about to give birth, i have no time to say a word. I often rush out all at once.” [Nina; Midwife]* |
|  | Lack of ability and awareness | 1. *“Sometimes after their termination, I don’t know what to say to them, and I don’t know how to comfort them. Because I have not experienced. I just comfort her not to be sad. There is no much deeper comfort.” [Kate; Midwife]* |
|  | Emotional experience of nurses and midwives | 1. *“You might be sympathetic when you first started working. But over time, you may become numb and less empathetic.” [Maria; Midwife]* |
| Training needs | Clinical professional knowledge and skills | 1. *“It is necessary for nursing staff to learn relevant medical knowledge, not necessarily as in-depth as the doctor, but we need to give a general idea and let women to discussion with doctors for specific details. Therefore, women will increase trust to you.” [Chloe; Nurse]* |
|  | Humanistic care skills | 1. *“I would like to learn more about the psychological characteristics of this population, because I have not studied relevant knowledge systematically and comprehensively. I want to know psychological responses in different stages so that i can use corresponding communication approaches. Another is the specific requirements that should be paid attention to when communicating with this population. There are no clear regulations and we have not received any training” [Emma; Nurse]* |
| Suggestions for benign interactions | Improvement of the medical environment and management | 1. *“Because there was no delivery room for me, I was outside all the time. The baby was left outside all the time. People who passed by would take a look at it, and it was a really bad feeling. It would be better if i could have a private room.”[Jane; Women]* |
|  | Optimization of bereavement care | 1. *“Some women know the baby is not alive. When she is going to give birth and the baby will come out soon, if there is no a medical staff in her side she will feel very afraid. In the future, we should strengthen the company of this population instead of leaving them alone.”[Sarah; Midwife]* |
|  | Preparing competent nursing professionals | 1. *“You may not be able to directly apply what you have learned from books, but you can't forget what you have experienced after reflection of clinical experience and then apply it to the clinic. It will accumulate more and more experience.”[Claire; Midwife]* |
